# Lean Library

**DOI:** 10.5195/jmla.2020.976

**Published:** 2020-07-01

**Authors:** Meghan Hupe

**Affiliations:** 1 maw55@georgetown.edu, Dahlgren Memorial Library, Graduate Health & Life Sciences Research Library, Georgetown University Medical Center, Washington, DC

## Abstract

**Lean Library.** Delft 48, 2611 CD Delft, Netherlands; https://www.leanlibrary.com/; three modules are available under the umbrella product of Lean Library: Library Access, Library Assist, and Library Alternatives; pricing is based partially on the number of modules implemented; libraries should contact the vendor for pricing.

## OVERVIEW

Lean Library “is a comprehensive tool that reliably and seamlessly extends the library resources, services and branding directly into the user's workflow” [1]. The tool is a browser extension that redirects users to legal open access versions of library-subscribed content or articles. One very important aspect of the Lean Library extension is that users do not need to start their research via the library's website as they are usually directed to now. Users can start from the publisher's website without needing to authenticate, and they can still access licensed content.

Lean Library simplifies a library's access to full-text resources and avoids unnecessary paywalls. The resource uses the Unpaywall application programming interface (API) to retrieve open access content and works with all major browsers, as well as Google Scholar, JSTOR, PubMed, and academic websites. When users search for an article, Lean Library connects to and searches their libraries' subscription holdings and provides access, or it “will detect the DOI (Digital Object Identifier) for an article and then validate with the library on whether the user has access through the library or another location. If the user has access at another location, the extension will offer to take the user there” [1]. When users are doing a book search, Lean Library “will identify an ISBN and check for eBook access. If the book is available electronically from the library, Lean Library will let users know with a helpful popup” [1].

The Lean Library icon is displayed as three squares on the toolbar and is greyed out when the tool is inactive. The icon turns green when users search for an item that is licensed by the library. When users click on the icon, the browser extension redirects them to the library's login page, where they are prompted to authenticate their credentials after which they can instantly access the content. If the library does not have access to the content, the tool automatically checks for open-access versions, and, if none are available, it generates a document delivery request through the library's interlibrary loan service. A yellow icon indicates that the institution has set up an important message about the item that users should be aware of.

Lean Library has a unique feature that allows users to highlight any text on a page, right click, and then select a specific search engine from the context menu. The Lean Library technical support team sets up the connection between the resource and the library search engine, which can include multiple options. At this reviewer's institution, the Dahlgren Memorial Library at Georgetown University, both PubMed and a beta discovery search tool are integrated with Lean Library. Users are directed to the library's resources that have information on the topic of interest.

Currently, with no mobile device access to Lean Library, it only works on desktops and laptops.

## GETTING STARTED

Lean Library setup is quite simple, but libraries will need to configure and maintain the product. For example, at Dahlgren Memorial Library, three months were set aside for a systems librarian to configure and roll out the product to patrons. Staff time and support prior to marketing, as well as ongoing maintenance, must be factored in. The Lean Library has three modules: Library Access, Library Assist, and Library Alternatives. The Library Access module simplifies access to subscribed resources; the Library Assist module provides messages to targeted users for information such as library workshops or temporary access issues; and the Library Alternatives module provides alternative legal routes to full-text articles.

When Lean Library is configured, users select a browser (all major browsers are supported) from Lean Library's website. After the extension has been downloaded, the icon will be displayed in the toolbar and will be greyed out until users are on a page with a citation or configuration has been completed. Users click on the icon, and the settings feature displays. Users are required to select their institutions and have the option to select an extra affiliation if they desire.

Users can also select “Skip the popup and automate my access” and “Show timer, when extension will close automatically.” The “Skip the popup and automate my access” function redirects users to their affiliated institutions' proxy access. The “Show timer, when extension will close automatically” function shows a timer in the pop-up display from users' institutions and can be paused if extra time is needed to review a resource. Users can change their settings at any time by clicking on the gear icon in the pop-up messages. The Welcome page provides an overview video where users can select different types of training on using this resource. Lean Library's Frequently Asked Questions section and email support team is available for further assistance.

## COMPARISON TO SIMILAR PRODUCTS

Several browser extension products are comparable to Lean Library.

- Kopernio is a browser extension available through Clarivate. Similar to Lean Library, Kopernio displays an icon that is integrated on the user's page that indicates whether an article is available. Kopernio is free; however, there is a fee for the premium version. This tool also has a feature that is referred to as a “locker,” where users can store articles in their accounts. Kopernio was reviewed in the October 2019 issue of the *Journal of Medical Library Association* [2].
- Unpaywall is a tool created by Impacstory that searches for open access journals. Similar to Lean Library, this extension tool has an icon located on pages that indicates if the article is available. Lean Library uses the Unpaywall API to obtain open access articles. Unpaywall was reviewed in the April 2019 issue of the *Journal of Medical Library Association* [3].
- LibKey Nomad is a fee-based extension developed by Third Iron and is only available on Google Chrome. Like the others, this tool has an icon located on each page to indicate accessible articles and verifies users' libraries' holdings.
- The Open Access Button and Google Scholar Button are both free and help users find open access articles via a toolbar icon.

## CONCLUSION

Lean Library is a browser extension that makes finding available content seamless. The “highlight any text on a page” feature is unique and offers users a quick way to access their libraries' content for further research. The Lean Library icon is located on the toolbar and provides an easy way for users to access content. Another benefit is that this tool provides security to the library's content because it requires user authentication with institutional credentials. Expanded access is an advantage thanks to the addition of the affiliated institution function. While Lean Library is subscription-based, its purchase improves the delivery of content in workflows; patrons do not leave the page to authenticate a journal subscription. Lean Library maintains a strong data privacy commitment, stating that no personally identifiable information is collected. Technical support is available through a Help Center link, chat windows, an administrative module, and email.
